# Remote Magnetic Nanoparticle Manipulation Enables the Dynamic Patterning of Cardiac Tissues

**DOI:** 10.1002/adma.201904598

**Published:** 2019-12-13

**Authors:** Limor Zwi-Dantsis, Brian Wang, Camille Marijon, Simone Zonetti, Arianna Ferrini, Lucia Massi, Daniel J. Stuckey, Cesare M. Terracciano, Molly M. Stevens

**Affiliations:** Department of Materials, Department of Bioengineering, Institute of Biomedical Engineering, Imperial College London, Prince Consort Road, London SW7 2AZ, UK; National Heart & Lung Institute, Imperial College London, The Hammersmith Hospital, Du Cane Road, London W12 0NN, UK; Department of Materials, Department of Bioengineering, Institute of Biomedical Engineering, Imperial College London, Prince Consort Road, London SW7 2AZ, UK; Department of Electrical and Electronic Engineering, Imperial College London, South Kensington Campus, London SW7 2AZ, UK; National Heart & Lung Institute, Imperial College London, The Hammersmith Hospital, Du Cane Road, London W12 0NN, UK; Department of Materials, Department of Bioengineering, Institute of Biomedical Engineering, Imperial College London, Prince Consort Road, London SW7 2AZ, UK; Centre for Advanced Biomedical Imaging, University College London, Gower Street, London WC1E 6BT, UK; National Heart & Lung Institute, Imperial College London, The Hammersmith Hospital, Du Cane Road, London W12 0NN, UK; Department of Materials, Department of Bioengineering, Institute of Biomedical Engineering, Imperial College London, Prince Consort Road, London SW7 2AZ, UK

**Keywords:** cardiac tissues, cellular organization, hydrogels, magnetic nanoparticles, patterning

## Abstract

The ability to manipulate cellular organization within soft materials has important potential in biomedicine and regenerative medicine; however, it often requires complex fabrication procedures. Here, a simple, cost-effective, and one-step approach that enables the control of cell orientation within 3D collagen hydrogels is developed to dynamically create various tailored microstructures of cardiac tissues. This is achieved by incorporating iron oxide nanoparticles into human cardiomyocytes and applying a short-term external magnetic field to orient the cells along the applied field to impart different shapes without any mechanical support. The patterned constructs are viable and functional, can be detected by *T*_2_*-weighted magnetic resonance imaging, and induce no alteration to normal cardiac function after grafting onto rat hearts. This strategy paves the way to creating customized, macroscale, 3D tissue constructs with various cell-types for therapeutic and bioengineering applications, as well as providing powerful models for investigating tissue behavior.

Directing cellular organization within 3D hydrogel matrices in a controlled manner is of great importance in the field of regenerative medicine and remains a major challenge. A multitude of technologies have been described to control the spatial organization of cells in 3D engineered heart constructs including mechanical strain/load^[1]^ and chronic electrical stimulation.^[2]^ Other approaches to guide cellular organization have been reported using microfluidic platforms,^[3]^ light-triggered activation of biomolecules,^[4]^ and 3D bioprinting.^[5]^ However, these techniques often involve elaborate, macroscale stimulation systems and are not always suitable for the fabrication of detailed microarchitectures in vitro as each pattern requires new molds, posts, or frames.^[6]^

The next generation of dynamic systems may be designed to respond to user-defined size and shape triggers for controlling cellular organization on the macroscale without the need for external mechanical supports or material cues. Magnetic procedures to manipulate and remotely control cellular behavior represent a promising approach for fabrication of tissue-like constructs. In particular, magnetic nanoparticles (MNPs) have gained increased attention for use in biomedical applications such as magnetic targeting of stem cells^[7]^ and genes,^[8]^ development of scaffold-free multilayer structures,^[9]^ and spatial patterning of aggregates.^[10]^ Magnetic techniques are advantageous due to their high precision and accuracy. To date, magnetic fabrication of biological structures has been illustrated by the assembly of biomembranes made of organized yeast,^[11]^ the formation of “artificial retinas” by magnetic field modulation of chiromagnetic nanoparticles,^[12]^ or the engineering of vocal folds,^[13]^ among others.

Here, we report a new platform for engineering tissue morphologies with controlled geometries. Specifically, we used magnetic fields to direct the assembly and patterning of magnetized human cardiomyocytes (CMs) labeled with MNPs in collagen-based hydrogels. Our system enables dynamic manipulation of cells within 3D biomaterials that can be applied to engineer patterned tissues to investigate cellular and tissue behavior. Furthermore, the simplicity and the faithful reproduction of our approach will enable the creation of customized 3D constructs with a new range of complementary implementations such as in biomedical devices, soft robotics, and flexible electronics.

First, we designed functionalized MNPs to target and label human induced pluripotent-stem-cell-derived cardiomyocytes (hiPSC-CMs, Figure 1a). For that purpose, we conjugated an anti-signal-regulatory protein alpha (SIRPA) cell surface monoclonal antibody^[14]^ labeled with a fluorophore to three types of MNPs with different core diameters and coatings: carboxyl terminated iron-oxide particles with 20 nm core diameter, and protein G conjugated MNPs with 100 and 500 nm core diameters (Figure S1a, Supporting Information). We then confirmed the ability of the functionalized MNPs to bind to human heart cells by Prussian blue iron staining (Figure 1b), confocal imaging (Figure 1c) and wide-field microscopy (Figure S1b, Supporting Information). In addition, we observed no significant impairment of CMs viability compared to the control-unlabeled CMs as assessed by measuring the cells’ metabolic activity over 10 days after labeling (Figure 1d). Hereafter, we selected to focus on the 20 nm core diameter SIRPA–MNPs due to their superparamagnetic properties, and their good dispersibility within the CMs that allowed better manipulation of the cells without the particle aggregation observed with the 100 and 500 nm MNPs (Figure 1b).

Compared to the free, nonconjugated MNPs, the 20 nm MNP–SIRPA had a slightly larger hydrodynamic diameter (Figure S2a, Supporting Information), and lower negative zeta-potential (Figure S2b, Supporting Information). A high conjugation efficiency was confirmed by the enhancement of the nanoparticles’ fluorescence intensity post-conjugation (Figure S2c, Supporting Information). To quantify the cellular MNPs uptake, we assessed the percentage of Cy5.5 positive cells at different MNP concentrations (20–50 μg mL^−1^) 24 h post labeling by flow cytometry and found that the SIRPA–MNP effectively labeled hiPSC-CMs (Figure S2d, Supporting Information).

To further characterize CMs labeling with the MNPs, we performed transmission electron microscopy (TEM) imaging at four time-points after cell labeling. As depicted in Figure 1e, the SIRPA–MNP were all internalized and localized within lysosomes. The TEM images also indicated that the CMs remained labeled with the MNPs for at least 21 days after their administration. This is in accordance with the CMs phenotype that lacks proliferative capacity,^[15]^ and therefore is expected to remain magnetized for longer time periods compared to dividing cells.^[16]^

Next, we aimed to demonstrate our newly developed approach that enables remote control of the magnetized CMs organization and distribution within 3D hydrogels by external magnetic fields. To this end, we created three different microarchitectures of cardiac tissues by exposing the cardiac cells to different shapes and sizes of neodymium (NiCuNi) permanent magnets (Figure 2a,b; Figure S3, Supporting Information). First, the magnetically labeled cells were mixed in collagen type I liquid suspension (at final concentration of 2 mg mL^−1^) and seeded in the center of a glass-bottom 35 mm plate above two ring-shaped permanent magnets (attached together). The applied magnetic fields (190–240 mT, Figure 2c,d) made the magnetized CMs reorder along the field direction during the gelation period and to form a ring-shaped contracting cardiac tissue (Figure 2e,f). The second magnetic arrangement was composed of opposing permanent magnets. A simulation of the magnetic field showed two high intensity peaks close to the center of the magnets where the magnetic field was calculated to be highest at around 200 mT. The mixture of MNP-labeled cells and collagen was placed in between the magnets on top of glass coverslip and the CMs migrated toward the magnets, forming a gradient of cells along the magnetic field where most of the cells concentrated close to the highest magnetic field (Figure 2g,h). For the third pattern, we used seven superimposed small circular magnets placed below the center of a glass-bottom 35 mm dish. In order to create a pattern of low and high cell densities within the same construct (herein defined as low/high constructs), we mixed CMs labeled with MNPs and unlabeled cells at a ratio of 1:1. Most of the magnetized cells were concentrated above the magnet, creating a high-density area of labeled cells in the center (where the calculated magnetic field was around 100 mT), and a low-density area of labeled cells in the surroundings together with the unlabeled cells which are not attracted to the magnet (Figure 2i,j).

In all cases, once the external magnets were removed, the cells remained embedded in the collagen hydrogels and maintained their induced orientation in the 3D form for at least two weeks. In the absence of magnetic field, the CMs were homogenously dispersed within the hydrogel as observed by the green fluorescence signal (Figure 2k–m).

Overall, the size of the ring-shaped and low/high constructs was 6 mm in diameter, while the gradient hydrogel was ≈10 mm in diameter. It is anticipated that larger constructs can be assembled by increasing the number of cells and by using large-sized external permanent magnets, respectively. Of note, the ability to manipulate the cells within other hydrogel types (such as fibrinogen, alginate, or gelatin) may also be possible and could be the focus of future studies. The choice of hydrogel should not influence the final configuration that is predominantly determined by the shape of the external magnets.

Next, we confirmed these results with numerical simulations of cell distribution performed with the commercial software COMSOL Multiphysics. Different stages of the migration are displayed in Figure S4 and Movies S1–S3 in the Supporting Information. In all cases, the dynamics are reproduced with good accuracy, leading to the same final distribution of labeled and unlabeled cells. COMSOL software was then used to further evaluate the magnitude of the intracellular magnetic forces which attract the cells to each other while migrating along the external magnetic fields. From the plots depicted in Figure S5 in the Supporting Information, we can conclude that these intercellular forces are at least one order of magnitude smaller than the applied external magnetic force and therefore are negligible.

Taken together, these findings show that the cellular loading with MNPs and the relative low external magnetic field applied (0.1–0.2 T) are sufficient to drive the cardiac cells along the field lines and pattern them to the desired controlled geometries within the collagen hydrogels. When compared to the high magnetic fields applied in medical applications (1.5–3 T in magnetic resonance imaging (MRI) scans^[17]^), the cells in our setup are exposed to 10–30 times lower static magnetic strength for a short term. Under these conditions no harmful effects to the cells were observed, and viable and functional cardiac tissues were formed. Notably, within 48–72 h after assembling the cardiac tissues with the magnetized CMs either with or without (control) magnetic field application, we could already detect synchronous mechanical activity (Movies S4 and S5, Supporting Information), and the cells continued beating in the hydrogels for several weeks. We then focused on the patterned hydrogels that are more relevant to cardiac field—the ring-shaped and the low/high density engineered constructs—for detailed structural characterization.

Immunostaining studies revealed that the CMs within the beating-labeled hydrogels were arranged in a typical striated isotropic pattern as confirmed by positive staining for sarcomeric *α*-actinin (Figure S6a, Supporting Information). In addition, we identified punctuate connexin43 (Cx43) immunosignal, suggesting the development of gap junctions (Figure S6b, Supporting Information). Interestingly, the cells in the ring-shaped pattern were organized and aligned along the orientation of the ring magnet as opposed to the unpatterned hydrogels where the CMs were organized randomly. This is consistent with previous studies showing that magnetic force can modulate F-actin dynamics and alignment.^[18]^ We could also identify cellular orientation in the area surrounding the high-density cells that could be attributed to the mechanical forces applied in the center of the construct due to the high number of CMs in this area (Figure 3a,b).

Finally, iron-oxide particles are known as medical imaging contrast agents as they attenuate magnetic resonance signals and result in negative contrast on MRI.^[19]^ Based on these properties, we demonstrated that *T*_2_*-weighted MRI can be used to visualize the structural properties of the magnetic hydrogels ex vivo (Figure 3c; Figure S7, Supporting Information), as well as to noninvasively locate the labeled hydrogels both ex vivo and in vivo after grafting them onto rat hearts (Figure 3d,e).

Our system is unique in terms of its ability to control the condensation of cells and their spatial organization by external magnetic fields into the desired orientation. In other non-magnetic systems, densely packed cardiac tissues are formed mechanically by trapping the cells in casting molds with additional stretching devices.^[20]^ These approaches are laborious, may not be reproducible, and could lead to variation in the tissues formed. Moreover, the nonmagnetic techniques are limited in their capability to create various tissue geometries, and the macroscopic form of the final constructs is restricted to the shape of the casting molds.^[21]^ Through labeling the cells with MNPs and exploiting their tunable magnetic responsivity, we generated, to the best of our knowledge, the first functional 3D cardiac tissues in a remote magnetic-controlled architecture without additional external supporting structures. Compared with the nonmagnetic techniques, our platform offers additional advantages that include simplicity, high reproducibility, and improved robustness, as well as an attractive alternative to current methods to create organ-on-a-chip.^[22]^ The system we developed is also applicable to multiple other cell types, and therefore, can potentially be used to engineer tailored different microstructures of 3D constructs.

Another exciting possibility is to inject the MNP-labeled cells within the hydrogel as a liquid solution directly into the injured organ and then to apply external magnetic fields to orient the cells remotely during the gelation period to the desired shape according to the pathology need. Such an approach holds the potential for the design of different microstructure orientations in vivo for future tissue engineering therapeutic applications.

The presented method still has some limitations, including: 1) the cells should be mixed in the hydrogel in its liquid state and allowed to reach the desired orientation before it solidifies, 2) in our study, we used lower density collagen that enabled cellular migration and remodeling within the hydrogel construct.^[23]^ This concentration is commonly used for the development of 3D tissues for regenerative medicine as a tissue engineered substitute.^[24]^ However, the degree of the patterning may be impaired when using a stiffer collagen matrix (>10 mg mL^−1^). 3) Once the gel is formed, the shape is fixed and applying another magnetic configuration will not orient the cells to a different geometry.

Overall, the simplicity of our approach as well as the faithful reproduction of cellular organization in biomaterials will enable a new range of complementary implementations and model systems for cardiac regeneration. The remote actuation of cells in 3D scaffolds utilizing magnetic fields can be further applied in the future to many other complex tissue morphologies, organoid development, bioelectronic devices, and soft robotics.

## Experimental Section

Experimental details are available in the Supporting Information.

## Supplementary Material

Supporting Information is available from the Wiley Online Library or from the author.

Supplemental Movie 5

Supplemental Movie 3

Supplemental Movie 2

Supplemental Movie 4

Supplemental Movie 1

Supporting Information

## Figures and Tables

**Figure 1:**
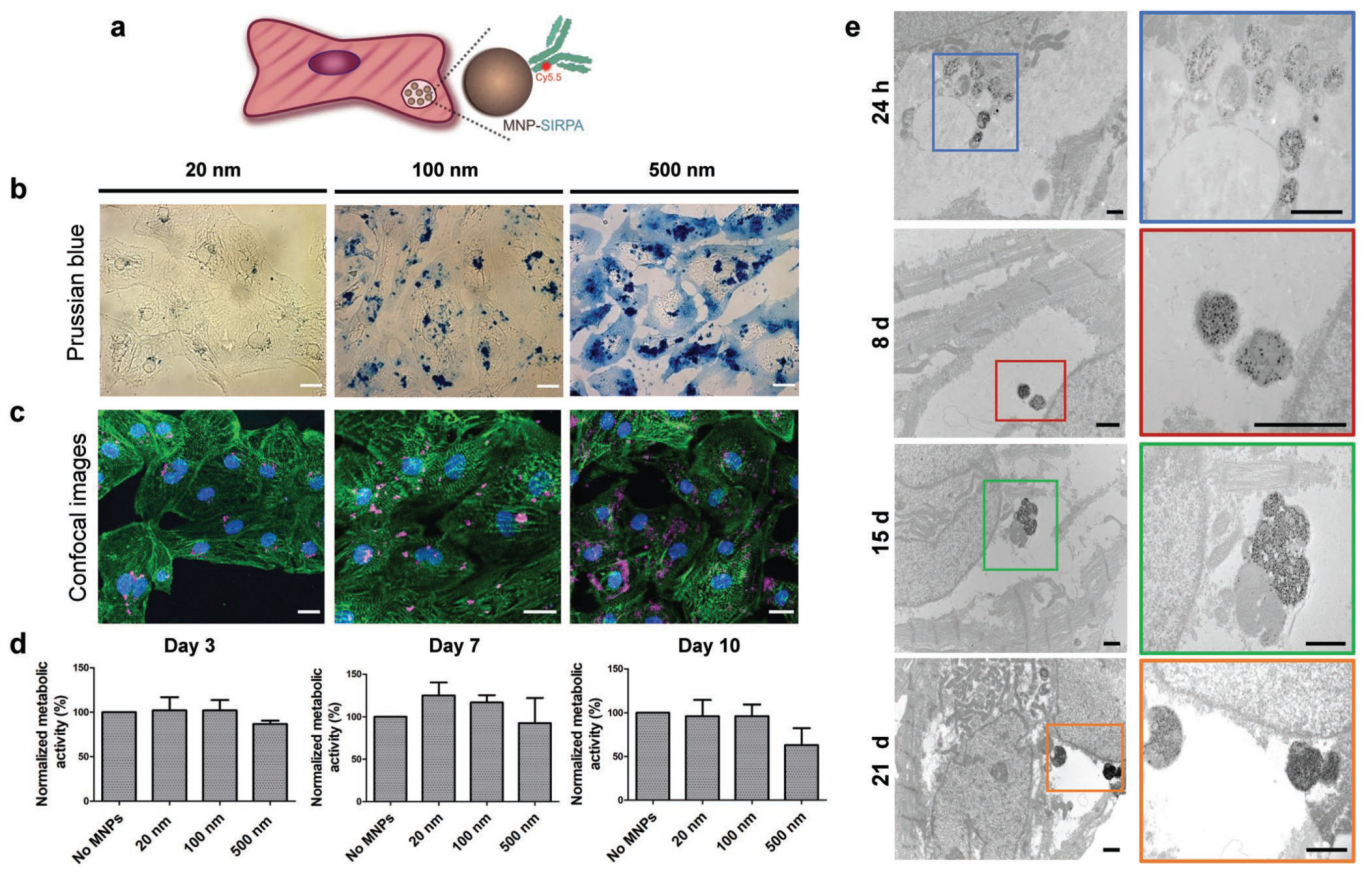
Targeting and magnetic labeling of hiPSC-CMs. a) Schematic view of the functionalized SIRPA–MNPs within the human cardiomyocytes. b) Prussian blue staining and c) confocal fluorescence images of the treated CMs 24 h after labeling with SIRPA–MNPs of different magnetic core sizes: 20 nm (left), 100 nm (middle), and 500 nm (right). Structural integrity of CMs was preserved upon MNPs loading (magenta), as observed by the distinct *α*-actinin staining (green); nuclei were stained with DAPI (blue). Scale bars: 20 μm. d) MTT metabolic activity assay at different time-points following 24 h incubation with SIRPA–MNPs. Data are shown as mean ± standard deviation (s.d.), *n* = 3 technical replicates, *N* = 3 independent experiments. No significant difference was observed between the labeled and unlabeled cells at all time-points (evaluated by one-way ANOVA followed by Tukey post-hoc test for day 3 and day 10 and Kruskal–Wallis test with Dunn’s multiple-comparison test for day 7). e) Transmission electron microscopy (TEM) images show MNPs internalization and accumulation within the hiPSC-CMs at different time periods post labeling. The squares show the magnified images of the corresponding particles. Scale bars: 1 μm.

**Figure 2:**
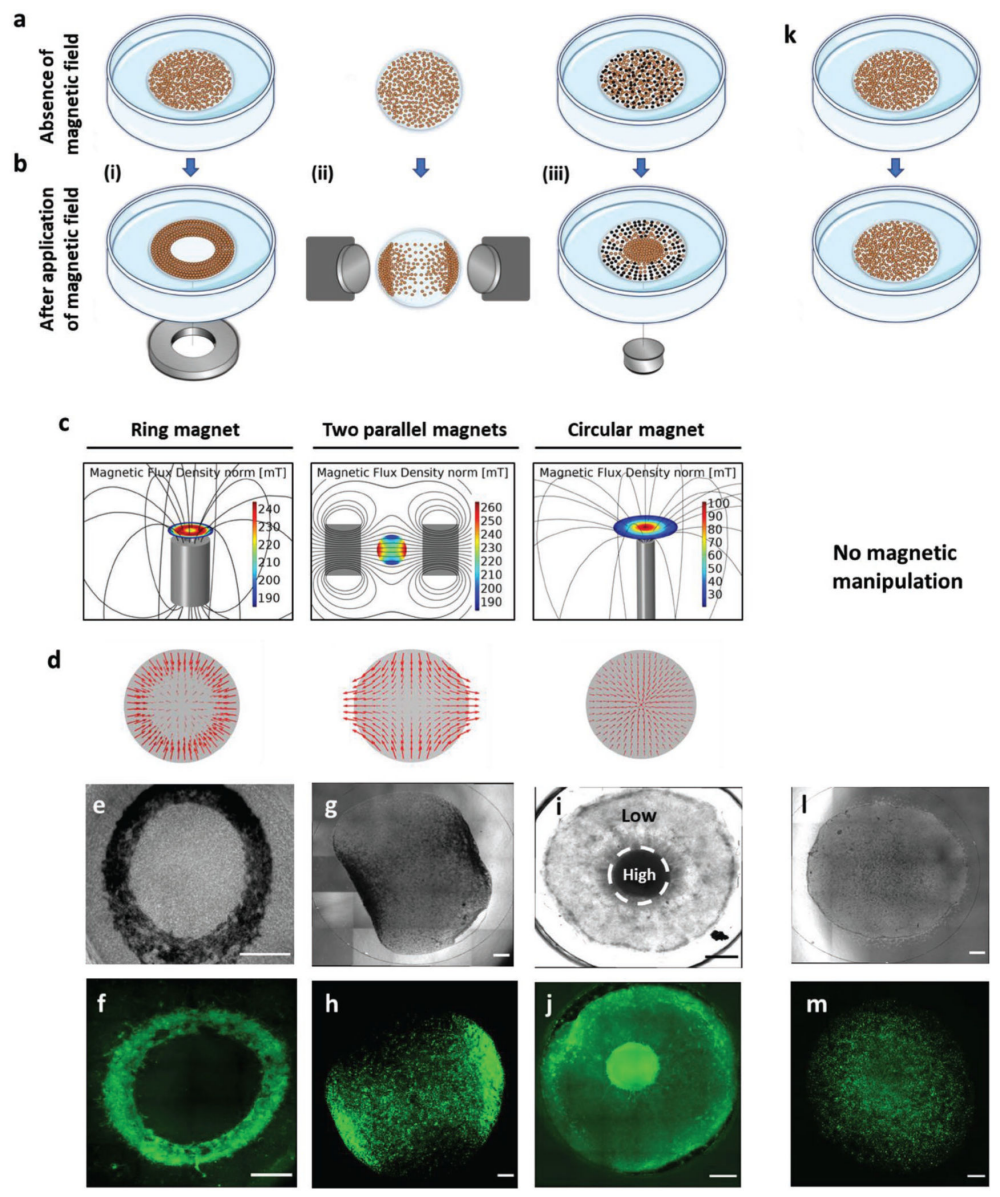
Engineering of patterned 3D human cardiac tissue constructs created by magnetic field manipulation. a,b) Schematic overview of the experimental setup. a) Liquid collagen suspension containing the hiPSC-CMs (labeled with MNPs—brown dots, or unlabeled cells—black dots) was placed in the center of a 35 mm glass-bottom dish or on top of a 13 mm glass coverslip; next, b) the cells were exposed to different shapes of external magnets: i) ring, ii) two parallel magnets, or iii) circular magnet and aligned along the direction of the magnetic field to create different patterned cardiac tissue constructs when the gels solidified. c) Simulation of magnetic flux generated by the different permanent magnet configurations in millitesla (mT). The direction of the magnetic field is indicated as solid lines and the intensity of the magnetic flux density norm at the dish surface is color coded (low intensity in dark blue, high intensity in red). d) The red arrows represent the field direction generated by the different magnetic setups in correspondence to the force distribution. e–j) Representative bright-field (top) and fluorescence (bottom) images of the patterned tissues in response to different magnetic field configurations: e,f) ring magnet, g,h) parallel magnets, and i,j) circular magnet, where high density of cardiac cells was formed in the center above the location of the magnet. k) Control hydrogels, where no magnetic field was applied, revealed random distribution of the cardiac cells within the hydrogel. l) Representative bright-field and m) fluorescence images of the control-labeled tissues. CMs were stained with *α*-actinin (green, ring and circular magnets) or with Calcein AM (green, parallel magnets and control). Scale bars: 1 mm.

**Figure 3:**
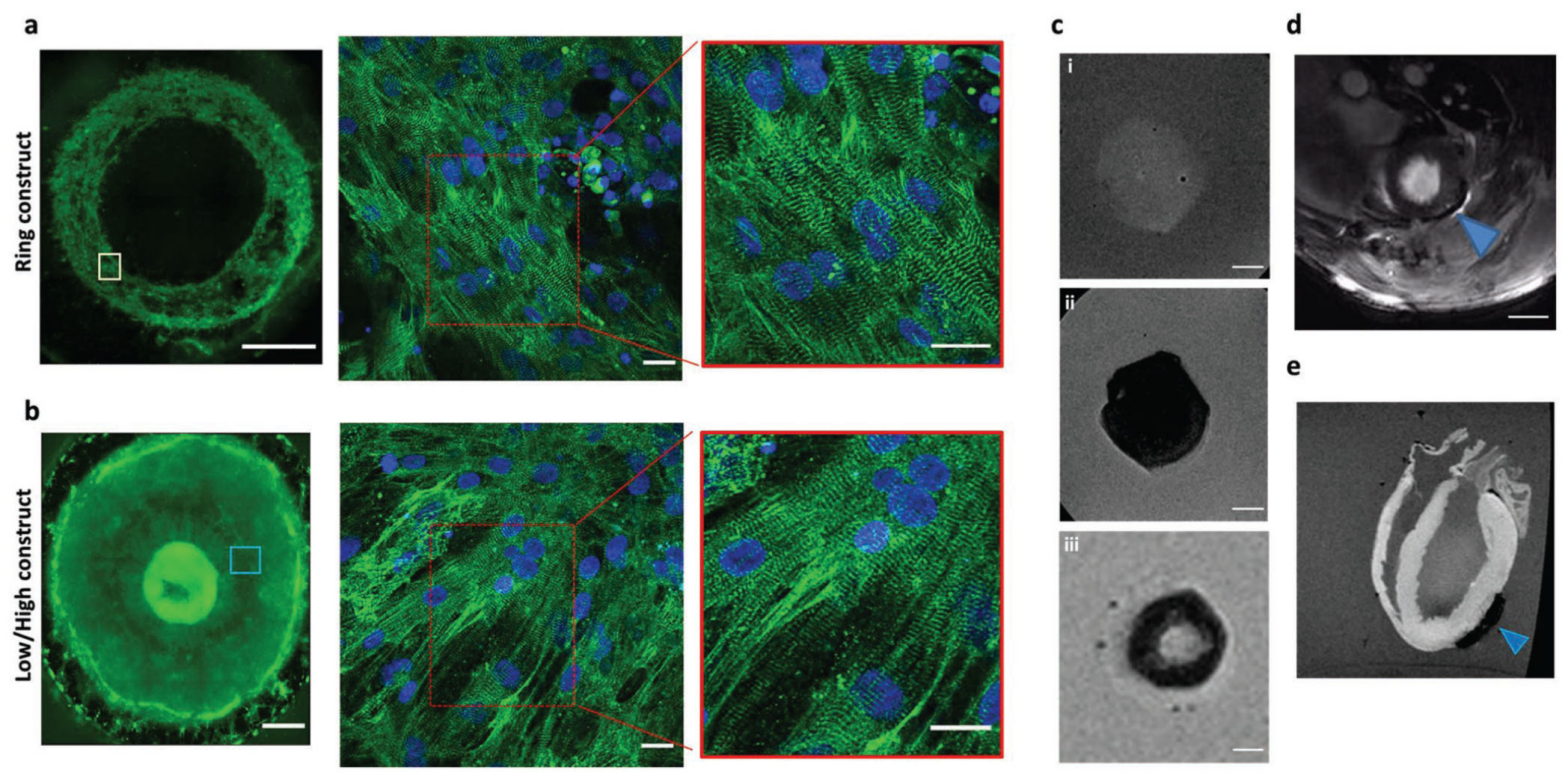
Structural characterization and MRI detection of the cardiac hydrogels. a,b) Immunostaining with *α*-actinin (green) revealed: a) the organization of the hiPSC-CMs within the ring-shaped hydrogel, and b) the low/high cell density hydrogel created by the circular magnets. Scale bars: 1 mm for the whole constructs; 20 μm for insets. Nuclei were stained with DAPI (blue). c) Ex vivo *T*_2_*-weighted MRI visualization of the different cardiac hydrogels: i) unlabeled tissue (control) had no signal void; while ii) the unpatterned labeled construct, and iii) the ring-shaped tissue caused a large signal void and could be detected by MRI. Scale bars: 2 mm. d,e) Representative in vivo (d) and ex vivo (e) MRI images of the unpatterned MNP-labeled cardiac hydrogels attached to the epicardium of rat hearts 2 and 8 days after implantation, respectively. The arrowheads indicate the cardiac hydrogel location. Scale bar: 5 mm.
