# Supplementary material for: Remote Magnetic Nanoparticle Manipulation Enables the Dynamic Patterning of Cardiac Tissues
Source: Adv Mater. Author manuscript; Available in PMC 2020 Feb 12. (PMC7015704; doi:10.1002/adma.201904598)
Supplement: Supporting Information [file EMS85361-supplement-Supporting_Information.docx]

Supporting Information

Remote Magnetic Nanoparticle Manipulation Enables the Dynamic Patterning of Cardiac Tissues

*Limor Zwi-Dantsis, Brian Wang, Camille Marijon, Simone Zonetti, Arianna Ferrini, Lucia Massi, Daniel J. Stuckey, Cesare M. Terracciano and Molly M. Stevens**

Advanced Materials

**
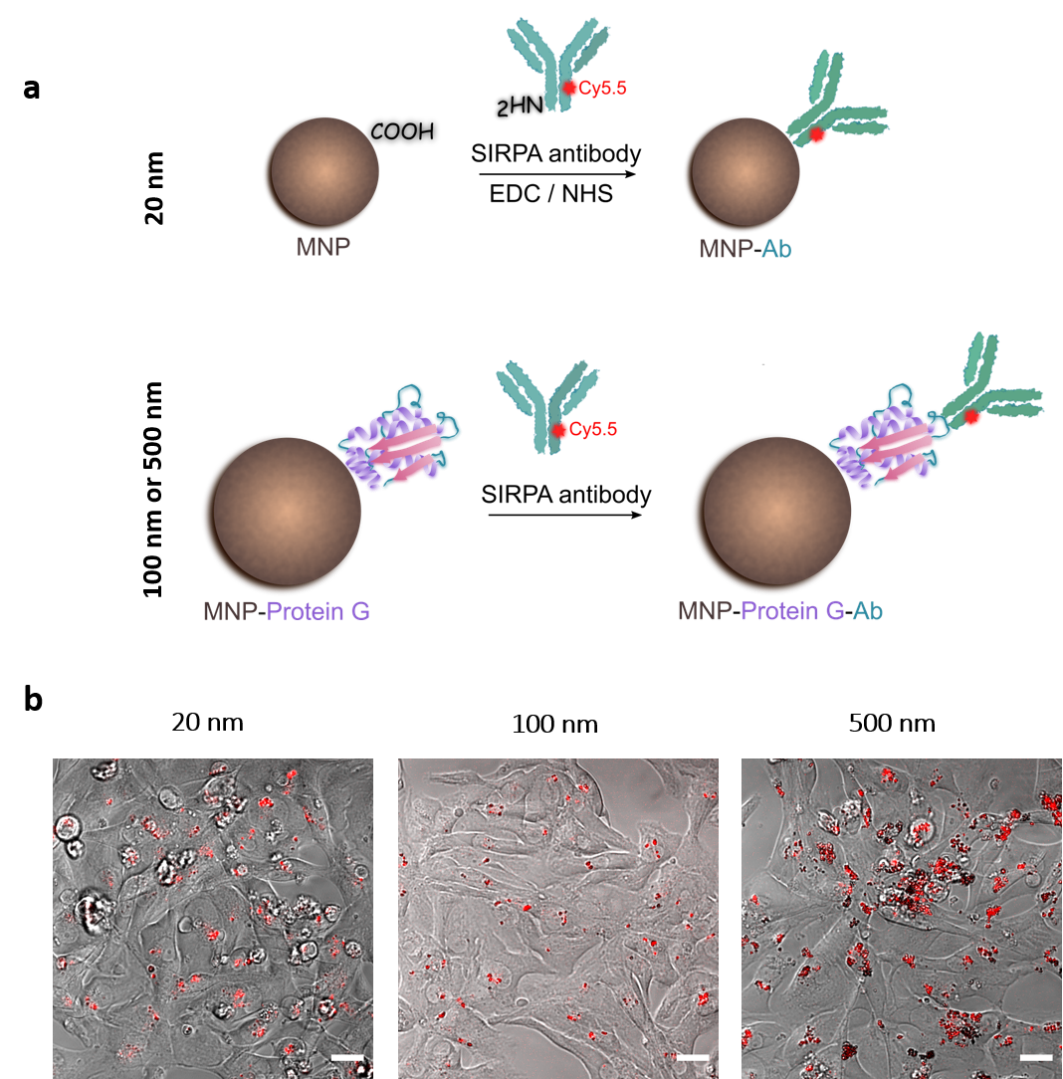
**

**Figure S1.** Magnetic nanoparticles functionalization and cellular uptake. **a**) Illustrations showing the conjugation routes used to produce the functionalized MNPs. The 20 nm carboxyl terminated iron oxide particles were conjugated to the SIRPA antibody through EDC/NHS coupling (top); in the 100 nm 500 nm particles, the antibody was linked to the protein G on their surface (bottom). **b**) Bright-field images of the cardiomyocytes 24 h after labelling with SIRPA-MNPs (red). Scale bars, 20 µm.


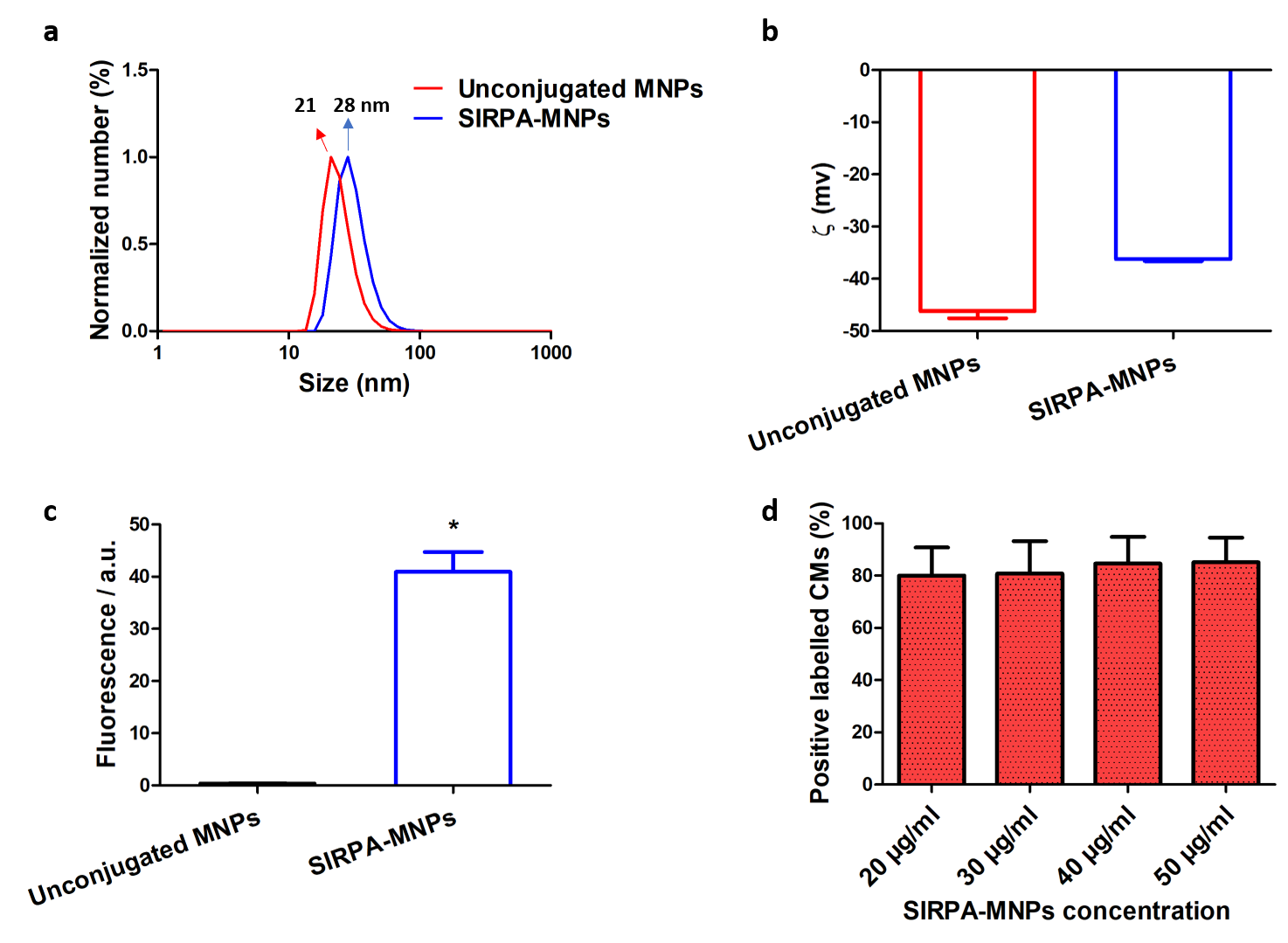


**Figure S2.** Characterization of the 20 nm MNPs and their cellular uptake. **a**) Dynamic light scattering (DLS) measurements for determining hydrodynamic size in the unconjugated- and SIRPA-MNPs. **b**) ζ-potential measurements of unconjugated MNPs and SIRPA-MNPs. **c**) Fluorescence signal intensity (emission/excitation = 482/690 nm) in the pre- and post-conjugation MNPs. Two-tailed unpaired t-test, *p = 6.64 x 10^-7^. **d**) Percentage of Cy5.5 positive CMs. Data shown as mean ± standard deviation ± s.d., *N = 4* independent experiments.


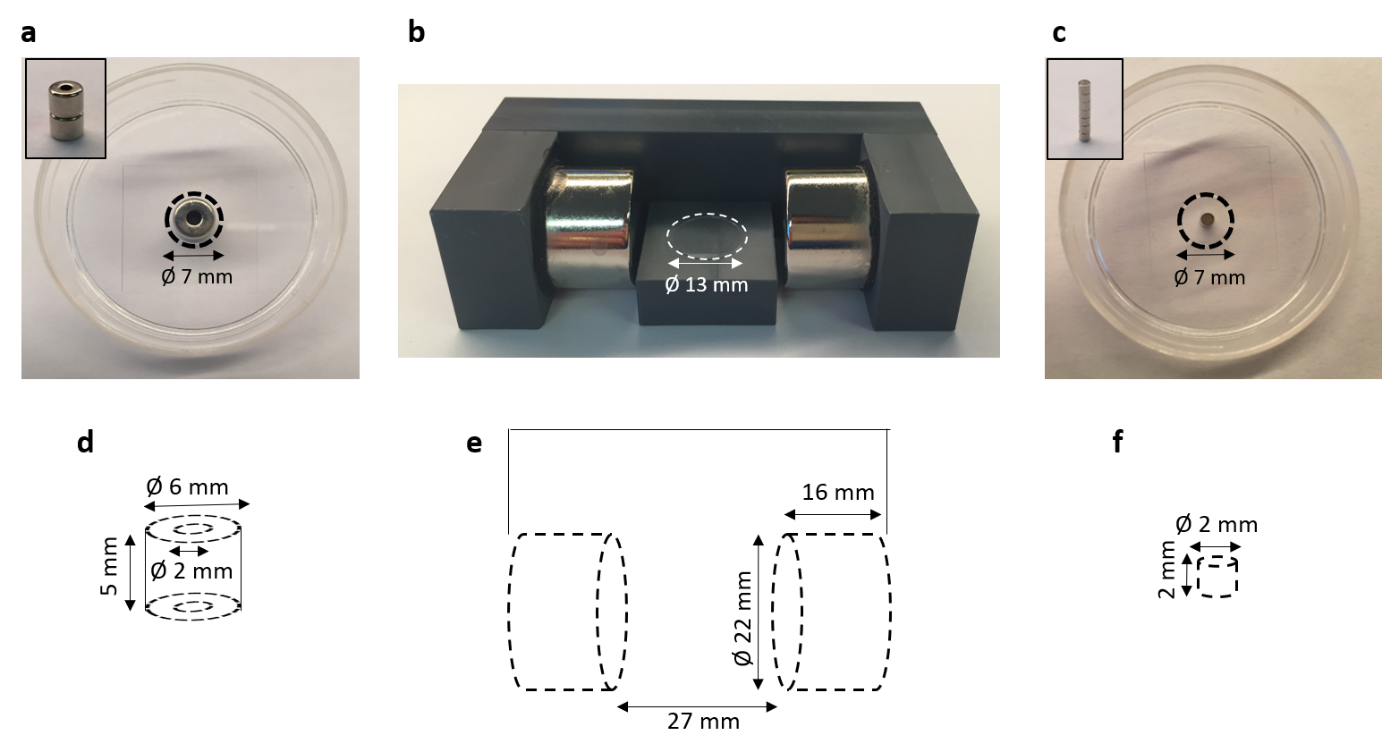


**Figure S3.** Magnetic stimulation set-ups. **a)** The magnetic device comprised of a holder containing two neodymium (NiCuNi) cylindrical magnets facing each other. A glass coverslip (white dash lines) is located between the magnets. **b-c)** Two ring magnets (**b**) and seven circular magnets (**c**) assembled together under a 35 mm dish in the center of the 7 mm diameter glass-bottom (marked in black dash lines). Insets: side-view of the magnets. The mixture of cells and hydrogel are placed on top of the coverslip (for the magnetic device) or on the glass-bottom area in the dish (for the ring and circular magnets). **d-f)** Schematic illustration of an individual magnet dimensions: cylindrical magnet in the magnetic device (**d**), a ring magnet (**e**) and a circular magnet (**f**).


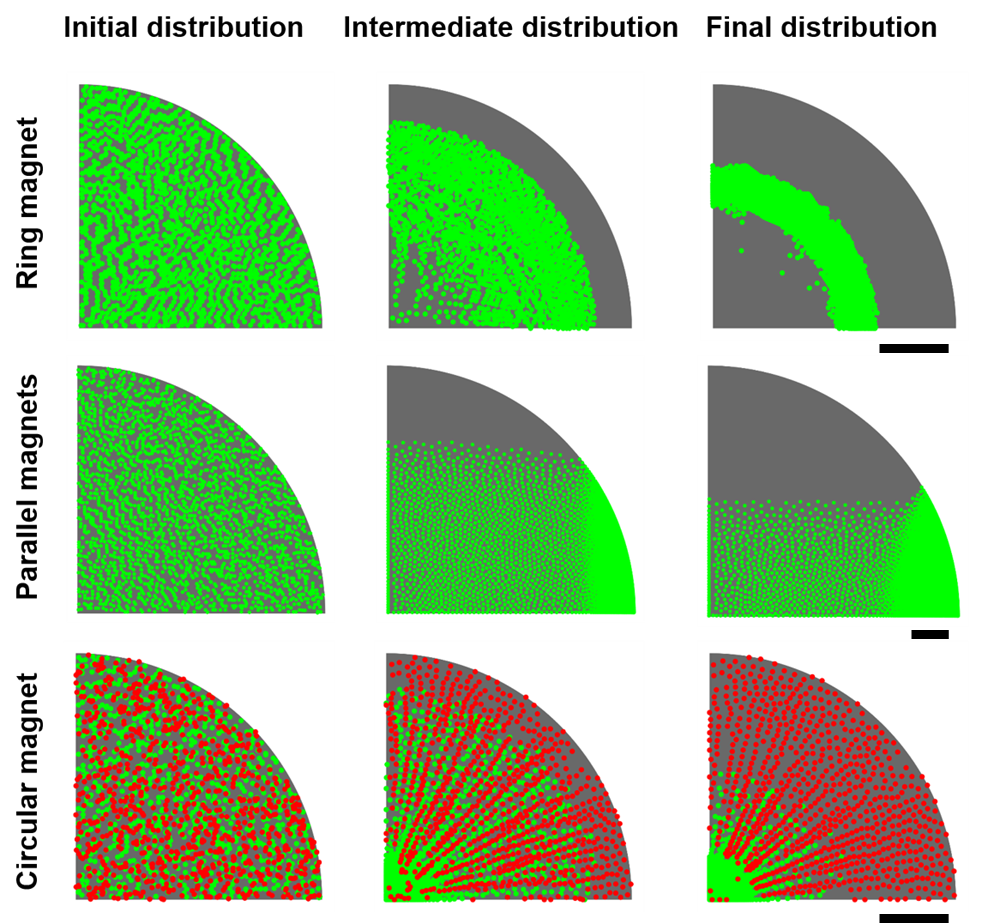


**Figure S4.** Simulations of cell migration within the hydrogels. COMSOL Multiphysics software was used to simulate the motion of point particles representing MNPs-labelled cells (green) or unlabeled cells (red). Time-sequence distributions are shown for each magnetic configuration. Scale bars, 1 mm.


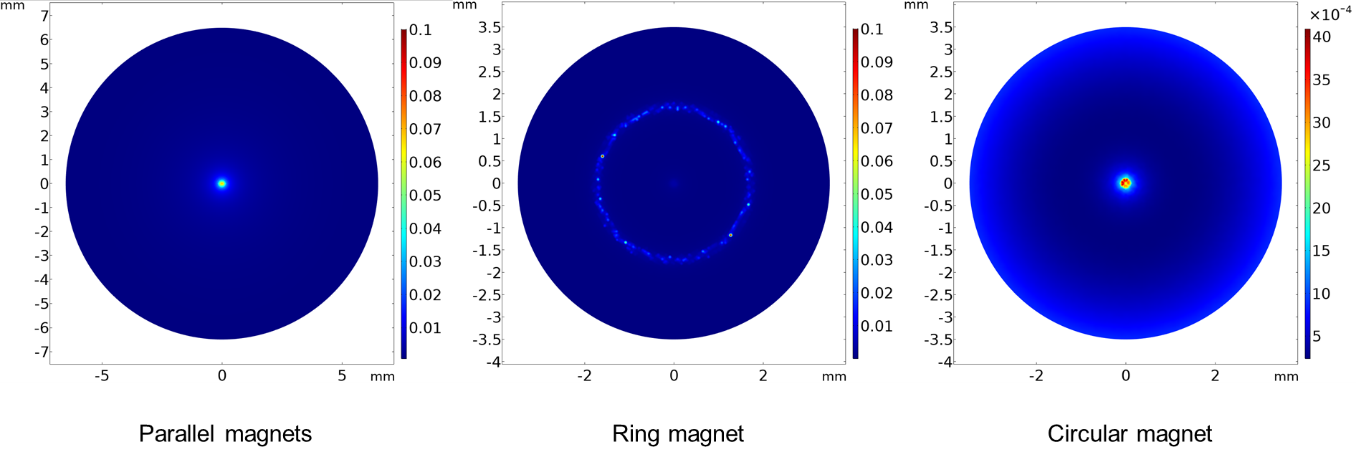


**Figure S5**. Simulation of the magnitude of intracellular forces at different magnet configurations. COMSOL Multiphysics software was used to evaluate the ratio between intercellular force and the external magnetic force (linear scale, right axes) for cells which are 75 µm apart. In all the magnetic configurations, the intracellular forces at least one order of magnitude smaller than the external magnetic force applied. Therefore, these forces are negligible, and the cells are attracted to desired orientation dominantly by the application of external magnetic fields.


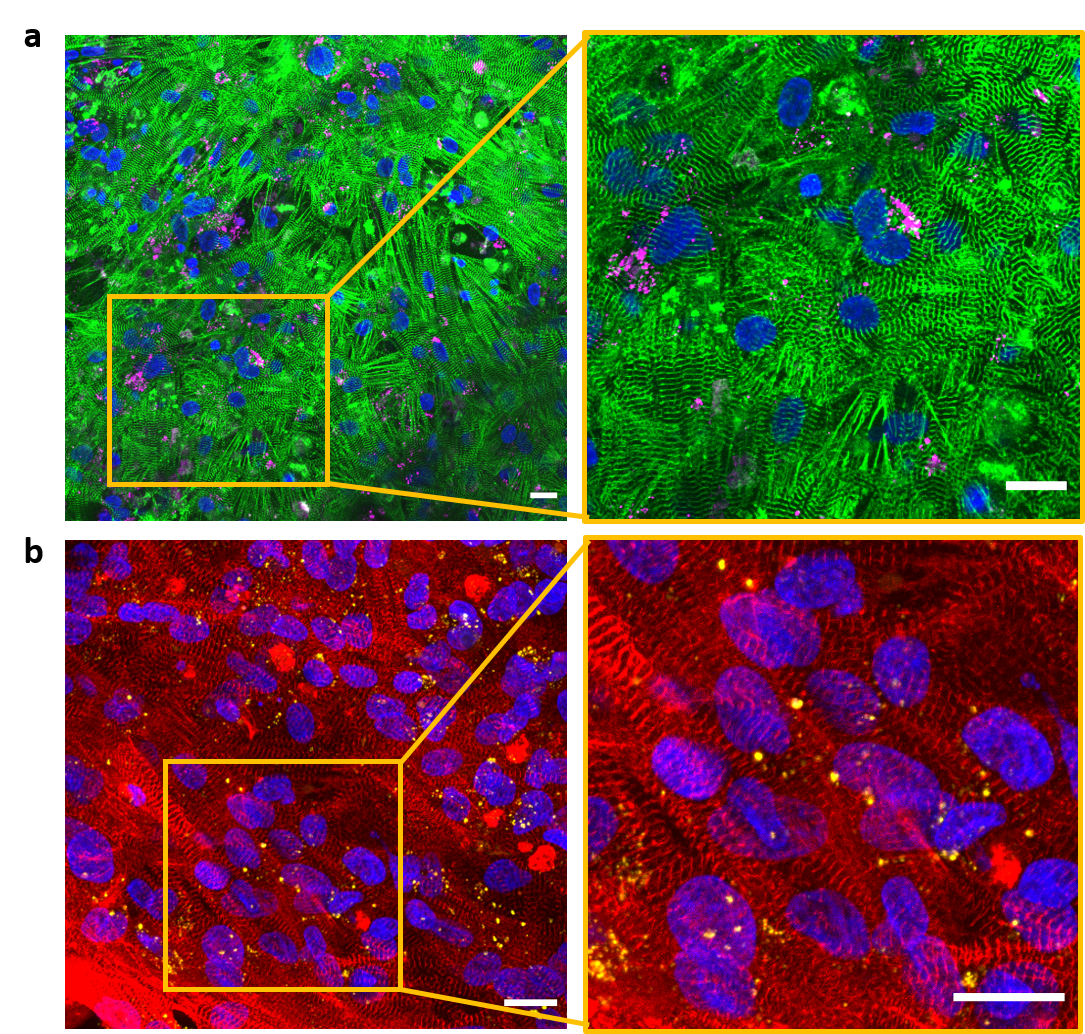


**Figure S6.** Structural characterization of the control MNP-labelled cardiomyocytes within the hydrogel without magnetic stimulation. **a**) Immunostaining for sarcomeric α-actinin (green) and the MNPs (magenta). **b**) Co-staining with sarcomeric α-actinin (red) and connexin43 (yellow punctuate staining). Nuclei were counterstained with DAPI (blue). Right panel: insets show high-magnification. Scale bars, 20 µm.


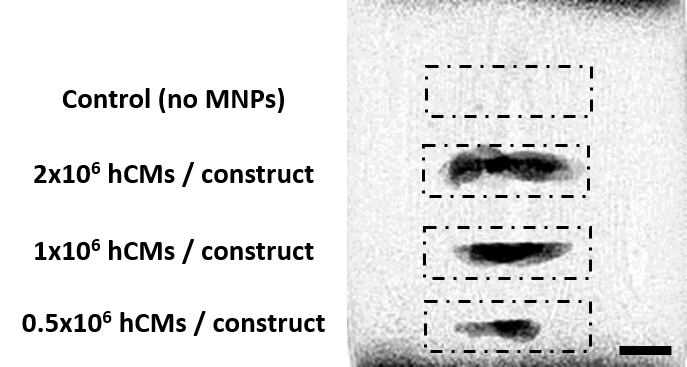


**Figure S7.** T2*-weighted MR images of the hydrogels at different cell concentrations. All the hydrogels containing MNPs-labelled human cardiomyocytes (hCMs) could be identified by a large signal void. No signal voids generated for the control non-labelled hydrogel (top row). Scale bar, 5 mm.

**Supplementary Movies**

**Movie S1.** COMSOL simulation - ring magnet

**Movie S2.** COMSOL simulation - parallel magnets

**Movie S3.** COMSOL simulation - circular magnet

**Movie S4.** Control-labelled beating hydrogel

**Movie S5.** Ring-shaped beating hydrogel

**Experimental Section**

*Culture of human induced pluripotent stem cells (hiPSCs) and cardiomyocyte differentiation:* Undifferentiated hiPSC colonies were cultured on 1:100 growth factor–reduced Matrigel (R&D Systems) using Essential 8 cell culture medium (Thermo Fisher Scientific). To passage the cells, 0.5 mM EDTA in D-PBS (Thermo Fisher Scientific) was used to dissociate the colonies for 7 min at room temperature every 3 – 4 days and re-plated in E8 medium supplemented with 2 µM of the ROCK inhibitor thiazovivin (Stratech Scientific) for 24 h following passaging. To induce differentiation, a differentiation medium, CDM3,^[1]^ was prepared, consisting of RPMI-1640 (Thermo Fisher Scientific), 500 μg mL^-1^ recombinant human serum albumin (Sigma-Aldrich), 213 μg mL^-1^ L-ascorbic acid 2-phosphate (Sigma-Aldrich), 1% (v/v) penicillin/streptomycin (100 U mL^-1^ and 100 g mL^-1^, respectively; Thermo Scientific). When the hiPSCs reached 80-90% confluence, the differentiation was started by changing the culture medium to a CDM3 medium supplemented with 6 μM CHIR99021 (tebu-bio) for two days. On day two, medium was replaced with CDM3 supplemented with 2 μM Wnt-C59 (Stratech Scientific) for two additional days. From day 5 onwards, the cells were cultured with CDM3 medium, and medium was changed every other day. On day 8 – 10, spontaneous contraction could be identified. To further increase cardiomyocyte purity, the differentiated cells were subjected to glucose starvation^[2]^ on days 10-16 post-differentiation. At day 10, the medium was changed to RPMI-1640 without D-glucose supplemented with 2% (v/v) B27 (Thermo Fisher Scientific) and 1% (v/v) penicillin/streptomycin for 3 days. At day 13, cells were returned to normal RPMI-1640 supplemented with 2% (v/v) B27 (RPMI/B27). At day 14, the medium was changed back to no-glucose medium for a second glucose deprivation cycle for an additional 3 days. At day 17 onwards, the cardiomyocytes were treated with RPMI/B27 medium (changed every other day). Flow cytometry was performed to ensure >95% cardiomyocyte purity. Dissociation of the beating monolayers into single cardiomyocytes was done enzymatically by applying TrypLE Express (Thermo Fisher Scientific) for 5 min at 37 °C.

*Iron oxide magnetic nanoparticles and conjugation of SIRPA antibody:* Three types of MNPs were used: carboxyl terminated iron oxide particles with 20 nm core diameter, and Protein G conjugated MNPs with 100 nm and 500 nm core diameters (all from Ocean Nanotech).

The 20 nm SIRPA-MNPs were generated by conjugating of PerCP/Cy5.5 anti-human SIRPA antibody (BioLegend) using the Carboxyl Iron Oxide Nanoparticle Conjugation kit (Ocean NanoTech) according to the manufacturer’s protocol. Briefly, 1 mg of MNPs were activated using 0.5 mg mL^-1^ of 1-ethyl-3-(3-dimethylaminopropyl)carbodiimide (EDAC) and 0.25 mg mL^-1^ sulfo-N-hydroxysulfosuccinimide (sulfo-NHS) in activation buffer for 30 min at room temperature with gentle rocking. Then, 50 µg of antibody were added to the activated MNPs together with coupling buffer to react for 2 h at room temperature with continuous mixing. At the end of the conjugation process, 10 µl of quenching buffer was added to the mixture for 10 min at room temperature. Then, the whole content was placed in a SuperMag Separator (Ocean NanoTech) to allow conjugated MNPs to separate at 4 °C overnight. The following day, the supernatant was removed and the resultant SIRPA-MNPs were washed twice with wash/storage buffer and stored at 4 °C in the same buffer.

To conjugate the SIRPA antibody to the protein G-MNPs, 1 mg of particles was mixed with 50 µg antibody in protein G IgG Binding Buffer (Thermo Fisher Scientific) for 1 h at room temperature with continuous rocking to allow the protein G to bind to the Fc region of the antibody through interaction with the heavy chain. The final SIRPA-MNPs were purified with a SuperMag Separator with wash/storage buffer and stored at 4 °C in the same buffer.

Conjugation efficiency was further assessed by fluorescence intensity measurements (excitation 480 nm, emission 690 nm) with a SpectraMax M5 Plate Reader (Molecular Devices). Hydrodynamic size and zeta potential were measured with a Zetasizer Nano ZS (Malvern Instruments).

*Prussian blue staining:* Prussian blue staining kit (Ocean NanoTech) was used to stain the MNP-labelled cells in blue. The cells were fixed in 4% (v/v) paraformaldehyde (Sigma-Aldrich) in D-PBS for 20 min at room temperature, rinsed three times with D-PBS and stained according to the manufacturer’s instructions. The samples were observed by a wide-field microscope (Zeiss Axio Observer).

*Immunostaining studies:* Single cardiomyocytes or collagen hydrogels were fixed with 4% (v/v) paraformaldehyde for 20 min at room temperature, rinsed three times in D-PBS, permeabilized with 1% (v/v) Triton X-100 (Sigma-Aldrich) for 10 min at room temperature, blocked with 5% (v/v) horse serum (Sigma-Aldrich) for 2 h in room temperature, and incubated overnight at 4 °C with primary antibodies, including connexin-43 (Cx43; 1:20, Santa Cruz) and sarcomeric α-actinin (1:200; Sigma-Aldrich). The following day, the preparations were rinsed three times in D-PBS and incubated with secondary antibodies: donkey anti-mouse immunoglobulin G (IgG) or donkey anti-goat IgG (all from Stratech Scientific) at a dilution of 1:200 for 1 h at room temperature. Primary and secondary antibodies were diluted in D-PBS containing 3% (v/v) horse serum. For immunostaining of the cells within the hydrogels, PBS-T (PBS + 0.1% (v/v) Tween 20) was used instead of D-PBS. Finally, samples were washed in D-PBS, mounted with Vectashield Antifade Mounting Medium (Vector laboratories) and nuclei were counterstained with DAPI (4’,6-diamidino-2-phenylindole) at 1:1000 (Sigma-Aldrich). Samples were examined with a laser scanning confocal microscope (Leica SP5). The images of the collagen hydrogels shown in Figure 4 were acquired using a Zeiss LSM-780 confocal microscope.

*Cell viability assay:* MTT (3-(4,5-Dimethylthiazol-2-yl)-2,5-diphenyltetrazolium bromide) assay (Thermo Fisher Scientific) was used to assess the cytotoxicity of the iron oxide nanoparticles on hiPSC-CMs. 1x10^4^ dissociated cells were seeded on 96-well plates, exposed to MNPs for 24 h, and then washed twice in D-PBS and replaced with culture medium. MTT assay was carried out at days 3, 7, and 10 following incubation with the MNPs. 10 µl of MTT stock solution (12 mM) was added to the 90 µl of RPMI/B27 culture medium (without phenol red) and incubated for 4 h at 37 °C. The formed formazan crystals were dissolved in 50 µl of DMSO (Sigma-Aldrich) for 10 min at 37 °C, and the absorbance at 540 nm was then measured using a SpectraMax M5 Plate Reader (Molecular Devices). Values were normalized relative to control values (unlabelled CMs) obtained under similar conditions.

*Flow cytometry analyses of cellular uptake of nanoparticles:* 24 h post incubation with MNPs, the hiPSC-CMs were dissociated with TrypLE for 5 min at 37 °C, incubated with a fixable viability dye eFluor 450 (eBiosciences) for 30 min at 4 °C, washed in D-PBD, filtered using 40 µm cell strainer (Falcon) and fixated using 4% (v/v) paraformaldehyde in D-PBS. Analytical flow cytometry was performed using LSR Fortessa I flow cytometer (BD Biosciences), and analysis was carried out through BD FACSDiva software (BD Biosciences).

*Magnetic cell patterning within hydrogels:* For magnetic cell labelling, 2×10^6^ cardiomyocytes were incubated with 40 µg mL^-1^ of 20 nm SIRPA-MNPs in RPMI/B27 culture medium for 18 h at 37 °C. After incubation period, samples were washed with D-PBS three times and then returned to RPMI/B27 medium.

Three different magnets were used: (1) the magnetic device was comprised of two N38 NiCuNi cylindrical magnets with 22 mm diameter and length of 16 mm, placed in a holder made of polyvinyl chloride (PVC, RS components) with 2.7 cm gap distance between two magnets; (2) N42 NiCuNi ring magnets with 6mm diameter, length of 5 mm and 2 mm diameter hole; and (3) N42 NiCuNi circular magnets with 2 mm diameter and length of 2 mm (all magnets from e-Magnets, UK).

For fabrication of the patterned cardiac hydrogels, 1×10^6^ MNP-labelled cardiomyocytes (or unlabelled cells as a control) were dissociated with TrypLE for 5 min at 37 °C, centrifuged at 200 g for 5 min and resuspended in 50 µl of Cardiomyocytes Maintenance Medium (CMM, Cellular Dynamics). The cardiomyocytes were then mixed with 70 µl of neutralized rat tail type I collagen (Corning) to a final concertation of 2 mg mL^-1^. Patterning protocols were adapted for each magnetic set-up: (1) for the ring and circular magnets – the mixture of collagen and cells was placed on 7 mm glass diameter coverslip within a 35 mm glass-bottom dish (MatTek). The center of the dish was then placed above two ring magnets (to form the ring-shaped construct) or above seven circular magnets (to generate the high/low density pattern); (2) for the magnetic device with two parallel magnets – the collagen mixture was seeded onto a 13 mm round coverglass (VWR) that was placed ± 0.7 cm from each magnet. Detailed schematic illustrations are shown in Figure 2a-b and Figure S3, Supporting Information. For all constructs, the cells within the hydrogel were patterned along the magnetic field during the slow gelation process of the hydrogel (50 min at room temperature) and were then removed from the magnets and maintained in CMM medium.

*Transmission electron microscopy (TEM):* The cardiomyocytes were dissociated with TrypLE, seeded on Nunc Lab-Tek Chamber Slide (Thermo Fisher Scientific) at a density of 1.5 ×10^5^ cells / 1.7 cm^2^, and cultured for 3 – 4 days to allow recovery of spontaneous beating activity. Then, the cells were incubated with SIRPA-MNPs for 24 h followed by three washes with D-PBS. The cells were either immediately fixed and stained for TEM imaging following established procedures^[3]^ or cultured in RPMI/B27 medium for an additional 8, 15, and 21 days before fixing and staining. The localization of MNPs on labelled cells was investigated using a Jeol 1200EX transmission electron microscope.

*Magnetic modelling:* For the static simulation of the magnetic field generated by the permanent magnets, the magnetic flux density was calculated by a Finite Element Analysis approach using the software COMSOL Multiphysics 5.3a (COMSOL AB, Stockholm, Sweden). For the dynamical time-dependent simulations of the cells migration, the Particle Tracing Module for COMSOL Multiphysics was used. An estimated magnetic momentum for the labelled cells was calculated in the order of 𝜇_𝑐𝑒𝑙𝑙_∼10^−10^𝐴𝑚^2^, with the magnetic field generated by the magnets considered to be static and independent of the distribution of the cells. A total of three external forces were considered: firstly, the magnetic force was calculated as the gradient of the scalar product between the magnetic momentum of the cell and the external magnetic field, with the assumption that cells will promptly orient their magnetic momentum along field lines. Secondly, the presence of the collagen was implemented with the presence of a drag force acting on moving cells, which is directly proportional to the viscosity of the collagen itself, and the diameter and the velocity of the cell. This provided resistance to the acceleration generated by the magnetic field. Thirdly, a repulsive force was introduced to model cell-cell interaction and ensure that cells do not penetrate each other. This force was taken to be proportional to the inverse of the square of the distance between cells, with a proportionality coefficient which is tuned according to the radius of the cells, expected to be around 100 µm. Computational constraints allowed the simulation of around 3500 particles, however the results can be considered valid for much larger numbers, given that the cell-cell interaction coefficient is tuned accordingly.

The magnitude of the intracellular forces was calculated as follows: $F_{cell-cell}=\frac{3}{4}\frac{m^{2}\mu_{0}}{\pi r^{4}}$ , where m is the magnetic momentum, r is the distance between cells, and µ_0_ is the permeability of the vacuum; while the magnetic force is given by: $F_{external}=\nabla(m\cdot B)$. Then, the ratio between the intercellular force and the magnetic force was calculated as: $\frac{F_{cell-cell}}{F_{external}}$.

*In-vivo hydrogel grafting and heart harvesting:* All animal procedures were carried out in accordance with the UK Home Office Animals (Scientific Procedures) Act 1986 and Directive 2010/63/EU of the European Parliament on the protection of animals used for scientific purposes.

Male Sprague-Dawley rats (250 – 350 g) were anesthetized with 5% isoflurane in O_2_ (Zoatis, UK), intubated with an appropriate cannula (Williams Medical Supplies, UK) and mechanically ventilated (Harvard Apparatus, UK). Body temperature was maintained using an adjustable heating mat. The hairs were removed from the left part of the thorax using clippers (Harvard Apparatus, UK) and the eyes were hydrated with Lacri-lube® (Allergan, UK). The surgical site was then scrubbed using diluted iodine solution, and isoflurane was then reduced and maintained at 2% during the whole procedure.

The heart was exposed by a horizontal incision of the skin followed by the dissociation of the thorax and intercostal muscles to access the base of the left ventricle. The hydrogel was sutured in 3–4 places to the epicardium with a non-resorbable 6/0 suture (Prolene, Ethicon, Belgium). The rib cage and muscles were then closed with a resorbable 4/0 suture (PDS™II, Ethicon, Belgium). The anesthesia was reduced to 1% isoflurane and the skin was closed with the same suture. At the end of the surgery, the anesthetic gas was turned off and the animal was extubated and placed in a heat chamber until full recovery. Animals were checked regularly for signs of pain/distress and analgesia was maintained for 1 day using buprenorphine (0.05 mg Kg^-1^, Vetergesic, Ceva, UK).

For heart isolation, the chest of the animal was opened to expose the heart. A solution of potassium chloride (50 mM in DI-H_2_O) was injected in the left ventricle through the apex to stop the heart in diastole, followed by a solution of 3.7% (v/v) paraformaldehyde (PFA, Sigma-Aldrich) to fix the tissue *in situ*. The heart was then removed, placed in 3.7% (v/v) PFA for 24 h at room temperature, and was then transferred to 70% (v/v) ethanol before preparation for *ex vivo* MRI and histology.

*3D MR microscopy:* The hydrogels or the excised rat hearts were fixed in 3.7% (v/v) paraformaldehyde (Sigma-Aldrich), and embedded in 1% (w/v) low-melt agarose (Bio-Rad). MRI was then performed at 9.4 Tesla using a Bruker Biospec MR system with a 39 mm birdcage radiofrequency coil. 3D gradient echo images were acquired with the following parameters TE/TR 1.8/15 ms, 15° pulse, field of view 32 × 32 × 32 mm, matrix size 128 × 128 × 128, voxel size 250 × 250 × 250 μm, six averages.^[4]^

*In vivo MRI:* Cardiac MI was performed as described previously.^[5]^ Rats were anesthetized with 2% isoflurane in O_2_ then transferred to a cradle which incorporates a rectal thermometer, heated water lines to maintain thermostasis (36.5 – 37 °C), a pressure transducer balloon to record respiration, three subcutaneous electrodes for recording ECG signals and a nose cone to deliver the anesthetic while the animal is in the scanner. Physiological monitoring was performed using an SA Instruments system (NY, USA). A 9.4 Tesla Varian MRI System was employed (Palo Alto, CA, USA) together with a 72 mm volume transmission coil with a 4-channel phased array surface coil for detection. Cardiac and respiratory-gated cinematic (cine) CMR imaging was performed in the true short-axis orientation and covered the whole left ventricle (LV) (1.5 mm slice thickness, TE/TR 1.6/5 ms: 17.5° ˚pulse, field of view 51.2 x 51.2 mm, matrix size 128 x 128, voxel size 400 x 400 x 1500 µm,25 to 35 frames per cardiac cycle, 3 signal averages).

*Statistical analysis:* All statistical analysis was performed using Prism software (GraphPad). Repeated-measurements one-way ANOVA followed by Tukey post-hoc multiple-comparison analysis was carried out for data with a parametric distribution, including comparison of cells metabolic activity (Figure 1d days 3 and 10). For data with a nonparametric distribution, Kruskal-Wallis test with Dunn’s post hoc analysis was used (Figure 1d day 7). Unpaired t-tests were used to compare ζ-potential and fluorescence signal intensity (Figure S2b-c, Supporting Information). Data are presented as mean ± standard deviation (s.d.) and p  < 0.05 was considered statically significant.

**Supporting Information References**

[1] P. W. Burridge, E. Matsa, P. Shukla, Z. C. Lin, J. M. Churko, A. D. Ebert, F. Lan, S. Diecke, B. Huber, N. M. Mordwinkin, J. R. Plews, O. J. Abilez, B. Cui, J. D. Gold, J. C. Wu, *Nat Methods* **2014**, 11, 855.

[2] A. Sharma, G. Li, K. Rajarajan, R. Hamaguchi, P. W. Burridge, S. M. Wu, *J Vis Exp* **2015**.

[3] J. R. van Weering, E. Brown, T. H. Sharp, J. Mantell, P. J. Cullen, P. Verkade, *Methods Cell Biol* **2010**, 96, 619.

[4] D. J. Stuckey, H. Ishii, Q. Z. Chen, A. R. Boccaccini, U. Hansen, C. A. Carr, J. A. Roether, H. Jawad, D. J. Tyler, N. N. Ali, K. Clarke, S. E. Harding, *Tissue Eng Part A* **2010**, 16, 3395.

[5] A. Chow, D. J. Stuckey, E. Kidher, M. Rocco, R. J. Jabbour, C. A. Mansfield, A. Darzi, S. E. Harding, M. M. Stevens, T. Athanasiou, *Stem Cell Reports* **2017**, 9, 1415.
